# Nanoemulsion Composed of α-Tocopherol Succinate and Dequalinium Shows Mitochondria-Targeting and Anticancer Effects

**DOI:** 10.3390/antiox12020437

**Published:** 2023-02-10

**Authors:** Le Thi Thuy, Seulgi Lee, Viet Dongquoc, Joon Sig Choi

**Affiliations:** 1 Department of Biochemistry, Chungnam National University, 99 Daehak-ro, Yuseong-gu, Daejeon 34134, Republic of Korea; 2 KM Science Research Division, Korea Institute of Oriental Medicine, Yuseong-daero 1672, Yuseong-gu, Daejeon 34054, Republic of Korea; 3 Department of Materials Science and Engineering, Chungnam National University, 99 Daehak-ro, Yuseong-gu, Daejeon 34134, Republic of Korea

**Keywords:** 3D spheroid, anticancer agent, dequalinium, nanoemulsion, tocopherol succinate

## Abstract

Targeted drugs have been used to treat mitochondrial dysfunction-related diseases, including metabolic disorders and cancer; however, targeting and penetrating intracellular organelles remains a challenge. Dominant targeting approaches for therapeutic delivery are detailed in many nanoemulsion studies and show the tremendous potential of targeted delivery to inhibit cancer cell growth. Dequalinium (DQA) and α-tocopherol succinate (α-TOS) are good agents for targeting mitochondria. In this study, we aimed to develop a mitochondria-targeting emulsion, using DQA and α-TOS (DTOS), for cancer treatment. DTOS emulsions of 150–170 nm in diameter were formulated using homogenization. DQA and α-TOS were used as bifunctional agents (surfactants) to stabilize the nanoemulsion and anticancer drugs. Various molar ratios of DQA and α-TOS were tested to determine the optimal condition, and DTOS 5-5 was selected for further study. The DTOS emulsion showed improved stability, as evidenced by its ability to remain stable for three years at room temperature. This stability, combined with its effective targeting of mitochondria, led to inhibition of 71.5% of HeLa cells after 24 h. The DTOS emulsion effectively inhibited spheroid growth in the 3D model, as well as prevented the growth of HeLa cells grafted onto zebrafish larvae. These results highlight the DTOS emulsion’s promising potential for mitochondria-targeting and cancer treatment.

## 1. Introduction

Mitochondria are energy-generating machines of the cells. They play a critical role in metabolism, which involves various vital cell pathway processes [1,2,3]. Many of these processes relate to diseases, such as cancer; therefore, carriers or drugs that target the mitochondria may be an efficient therapy. The main impediment in mitochondria therapy for cancer treatment is the effective transport of the anticancer drug to the mitochondria because of the dependence on the physicochemical properties of the drug, such as size, solubility, and interaction with the plasma, to overcome the mitochondrial membrane [4]. Therefore, the development of utilitarian nanomaterials is crucial in the field of nanomedicine.

Nano-sized systems such as liposomes, micelles, and nanoemulsions have been explored to deliver drugs [5,6,7,8]. Nanoemulsion is the heterogeneous dispersion of two immiscible liquids (oil in water or water in oil) at the nanoscale. Surfactants, compounds with an amphiphilic profile, are used to reduce the interfacial tension between two immiscible phases and stabilize the system [9]. The stability of nanomedicine is one of the requirements of drug delivery [10]. Previous studies showed that emulsions can be stored for around 3–6 months at room temperature [11]. The emulsions do not have a sufficiently long shelf-life and they present destabilization mechanisms such as creaming, sedimentation, coalescence, and flocculation [12]. Using a polyelectrolyte nano-capsulate layer-by-layer around the solid template can protect the emulsion [13]. The secondary emulsions oil–water–oil (O/W/O) and water–oil–water (W/O/W) have been applied to improve the stability of the emulsion [14]. Another approach is using a high-pressure homogenizer to prepare emulsions together with a food-grade polymer to stabilize O/W nanoemulsions; however, the final colloidal systems presented a wide size distribution, predictably leading to fast destabilization [15]. The merger of relevant techniques and surfactants can provide a stable emulsion. Previous studies have also highlighted that emulsions are able to achieve an efficient therapeutic effect, and a poor-solubility anticancer agent can be loaded into the oil phase, which can cover the droplet for cell- or organ-targeting [16]. Ganta developed a folate nanoemulsion to deliver docetaxel to target ovarian cancer cells [17]. Moreover, lycopene emulsions have been used to treat human colon cancer cell lines (HT-29) [18], a taxoid prodrug in fish oil emulsion was found to be effective against prostate cancer [19], and methotrexate emulsion was more effective for leukemia than the free drug was [20]. In addition, emulsions have been used to treat breast cancer, melanoma, and lung cancer [21,22,23].

Dequalinium (DQA) is an amphiphilic component that has been explored as a mitochondrial moiety [24]. DQA can form a vesicular structure because of its amphiphilic structure and was reported to induce apoptosis in U373-MG and HeLa cells [25]. Therefore, drug delivery systems modified with DQA offer great promise in future cancer therapies. Recently, DQA was combined with other lipid components to form mitochondrial-targeted liposomes [26]. DQA liposomes easily transferred the drug to the mitochondria and improved the effectiveness of the drug. DQA can be modified directly to the polymer for polymersome formulation. Glycol chitosan–DQA was developed for curcumin delivery [27]. Therefore, DQA is a good candidate for an emulsion system targeted at the mitochondria. However, there are no studies about the use of DQA in emulsions.

Among the vitamin E derivatives, α-tocopherol succinate (α-TOS) shows the most attractiveness in cancer treatment. α-TOS is well-known as an antioxidant component. α-TOS can cause reactive oxygen species (ROS) and lead to cancer cell death [28]. Cancer cells express the antiapoptotic Bcl-2 and Bcl-xL. Bcl-2 can bind onto Bax, and this enables the survival of cancer cells by inhibiting cell apoptosis. Tocopherol succinate is able to block the BH3 domain of the Bcl-2 and Bcl-xl complex. Therefore, it decreases the binding of Bcl-2 on Bax protein. Depending on that mechanism, α-TOS is sensitive to cancer cells. In addition, α-TOS is a coenzyme Q10 inhibitor because it can bind to complex II in mitochondria [29]. Coenzyme Q10 is an electron carrier in mitochondria. However, this process is inhibited by α-TOS, which leads to the release of electrons in the cell. These unpaired electrons react with cellular oxygen to form various reactive oxygen species, causing cell death. Therefore, using α-TOS can improve the effectiveness of cancer treatment. Tocopherol succinate emulsion for paclitaxel has an effect on the human ovarian carcinoma cell line A2780/taxol. Guo et al. [30] reported that α-TOS is a nonionic surfactant with anticancer effects. Tocopherol succinate can inhibit the multidrug-resistant efflux pump P-glycoprotein [31]. Tocopherol succinate also supports vitamin K to produce apoptosis in cancer cells [32].

**Figure 1:**
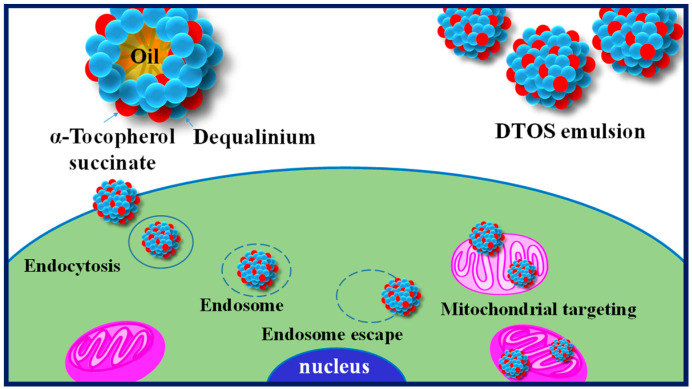
Illustration of the cellular uptake and mitochondria-targeting of the DTOS emulsion.

Harashima’s group has demonstrated the potential of lipid-based formulations as a means of delivering poorly water-soluble drugs, with mitochondria-targeting being a promising therapeutic strategy [33,34,35]. To this end, we developed a novel nanoemulsion system using DQA and α-TOS as emulsifiers (DTOS emulsion) and anticancer agents. Figure 1 shows the representation of the DTOS emulsion’s mechanism of cellular uptake and mitochondria-targeting. The high-pressure homogenization process was used to achieve mono-dispersion, and the combination of DQA and α-TOS was used to enhance stability. Various mole ratios of DQA and α-TOS were evaluated to determine the optimal conditions for encapsulation efficiency and stability. The DTOS 5-5 emulsion was selected for in-depth examination and its stability was found to be consistent for three years when stored at room temperature. The efficacy of its anticancer properties was also observed to be unchanged for three months. Treatment of HeLa cells with the DTOS emulsion resulted in increased cellular uptake and alteration of mitochondrial potential. This treatment led to over 70% inhibition of cell growth in cervical carcinoma (HeLa cells), breast cancer (MCF-7 cells), and murine melanoma (B16F10 cells). Furthermore, the DTOS emulsion demonstrated its anticancer potential in both 3D models (HeLa spheroid) and HeLa cells xenografted onto zebrafish larvae. These results suggest that the combination of DQA and α-TOS can be used to create a nanoemulsion for mitochondria-targeting in cancer therapy.

## 2. Materials and Methods

### 2.1. Materials

DQA chloride, castor oil, PEG-8 caprylic/capric glycerides, isopropyl myristate (IPM), lecithin, and N-(2-hydroxyethyl) piperazine-N′-2-ethanesulfonic acid (HEPES) were purchased from Sigma-Aldrich (St. Louis, MO, USA). Fetal bovine serum (FBS), 100× antibiotic-antimycotic agent and Dulbecco’s modified Eagle’s medium (DMEM) were purchased from GIBCO (Gaithersburg, MD, USA). HeLa, B16F10, and MCF-7 cell lines were obtained from the Korea Cell Line Bank (Seoul, Republic of Korea).

### 2.2. Control Emulsion

IPM, PEG-8 caprylic/capric glycerides, and lecithin were dissolved in a 20 mL bottle and sonicated for 15–20 min. Then, 10 mL of water was added, and the solution was sonicated again for 30 min to 1 h and homogenized for 2 h. Then, the solution was filtered with a 0.8 μm filter.

### 2.3. DQA Emulsion

DQA chloride 5 mM was dissolved in castor oil and sonicated for 15–20 min. Then, 10 mL of water was added, and the solution was sonicated again for 30 min to 1 h, and then the solution was homogenized. The solution was then filtered with a 0.8 μm filter.

### 2.4. DTOS Emulsion

**Figure 2:**
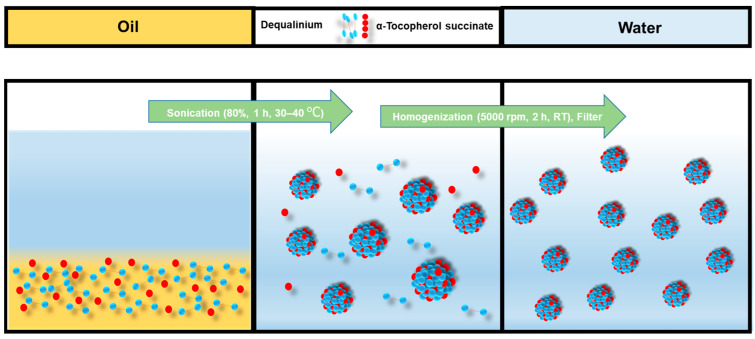
Emulsification process of DTOS emulsion.

DQA chloride and α-TOS were dissolved in castor oil and sonicated for 15–20 min. Then, 10 mL of water was added, and the solution was sonicated again for 30 min to 1 h, and then the solution was homogenized. The solution was then filtered with a 0.8 μm filter. The emulsion formulation process is shown in Figure 2.

### 2.5. Nile Red-Loaded Emulsion

DQA chloride, α-TOS, and Nile red were dissolved in castor oil and sonicated for 15–20 min. Then, 10 mL of water was added, and the solution was sonicated again for 30 min to 1 h, and then the solution was homogenized. The solution was then filtered with a 0.8 μm filter.

### 2.6. Absorbance

Absorbance of the emulsion was detected using a UV-Vis spectrophotometer (Optizen POP; Mecasys, Daejeon, Republic of Korea). Methanol was used as a solvent for DQA, and the absorbance was found to be 330 nm for DQA and 290 nm for α-TOS.

### 2.7. Fourier Transform Infrared Spectrum (FTIR) Spectroscopy

The IR spectra of the DTOS emulsion, DQA, α-TOS, and their mixture were recorded using the FT-IR Nicolet™ iS™50 spectrometer. The spectra were acquired as an average of 64 spectra with a resolution of 4 cm^−1^, and the dried state of the emulsion was placed into the holder for the analysis. The recording was performed in the spectral range of 600–4000 cm^−1^.

### 2.8. Size and Zeta Potential

The size of the emulsion was measured using a Photal ELSZ particle size analyzer (Otsuka Electronics, Tokyo, Japan). Measurements were performed in triplicate and evaluated as the z-average (size). The surface charge of the emulsion was measured using a Zetasizer Nano ZS (Malvern Panalytical, Malvern, UK) at room temperature.

### 2.9. Stability of Emulsion over Time

The emulsions were kept at room temperature or in a refrigerator, and the size was measured using DLS at certain times.

### 2.10. Cell Uptake of Emulsion

HeLa cells (8 × 10^3^ cells/well) were seeded in confocal dishes (µ-Slide 8 Well; ibidi, Fitchburg, WI, USA) and allowed to adhere in 270 µL of DMEM media (10% FBS and 1% (*w*/*v*) penicillin/streptomycin) at 37 °C in a humidified atmosphere of 5% CO_2_ for 24 h. Then, cells were incubated with 30 µL of Nile red emulsion for 24 h. The medium was removed, cells were washed with Dulbecco’s phosphate-buffered saline (DPBS) twice, and the nucleus was stained for 10 min with 200 µL of bisbenzimide (Hoechst 33342). The mitochondria were stained with 200 µL of MitoGreen (2 µM) for 20 min. Finally, cells were washed and observed using a Zeiss LSM 880 Live confocal laser microscope (Zeiss, Oberkochen, Germany).

### 2.11. Assessment of Mitochondrial Potential (ΔΨm) by JC-1 Staining

JC-1 (5, 59, 6, 69-tetrachloro-1, 19, 3, 39-tetraethylbenzimidazolcarbocyanine iodide) is a cationic dye used to target mitochondria. When a cell is healthy, JC-1 aggregated inside the mitochondria emit red fluorescence (590 ± 10 nm). However, when a cell is unhealthy, the mitochondrial potential is low, and the JC-1 monomers stay in the cytoplasm and emit green fluorescence (525 ± 10 nm). To assess the mitochondrial membrane potential of the DTOS emulsion, HeLa cells were seeded in confocal dishes (µ-Slide 8 Well; ibidi, Fitchburg, WI, USA) (8 × 10^3^ cells/well) and allowed to adhere at 37 °C under 5% CO_2_ for 24 h. Sample-treated (24 or 48 h) cells were then washed and incubated with 2 μM of JC-1 at 37 °C for 20 min. Cells were washed twice with DPBS, and analyzed using a Zeiss LSM 880 Live confocal laser microscope (Zeiss, Oberkochen, Germany).

### 2.12. Cell Viability

To determine the cell viability of the emulsions, a colorimetric WST-1 assay was performed. HeLa, B16F10, and MCF-7 cells were cultured in DMEM medium containing 10% (*v*/*v*) FBS and 1% (*w*/*v*) penicillin/streptomycin at 37 °C in a humidified atmosphere of 5% CO_2_. Then, 1.3 × 10^4^ cells/well were seeded in a 96-well plate and incubated in 90 µL of media for 24 h. Cells were then treated with 10 µL of DTOS emulsion along with the positive controls. After 24 h of incubation, 10 µL of EZ-Cytox reagent was added to each well (except for the negative control), incubated for 2 h, and the absorbance was measured at a 450 nm wavelength.

### 2.13. Anticancer Effect in 3D Spheroid Tumor Models

The anticancer effect of the DTOS emulsion was evaluated in 3D spheroid tumor models. The 3D spheroids were generated using a 1% agarose coating in a 96-well plate. Briefly, 0.2 g of agarose was dissolved in sterile distilled water and heated for 2 min in a microwave oven. Then, 100 μL of the homogenous agarose solution was quickly pipetted into the 96-well plate to coat a thin layer and left for 30 min to solidify. To form spheroids, 2 × 10^3^ cells/well were seeded in agarose-coated 96-well plates and left undisturbed for 24 h at 37 °C under 5% CO_2_. Spheroids were allowed to grow for 3 days, and samples were treated at different concentrations of DQA (0.6, 12.5, 25, 50, and 100 μM). The media in medium-containing samples were replaced every alternative day and spheroid growth was monitored. A spheroid without any treatment was used as a control.

### 2.14. Anticancer Effect in Zebrafish Models

Wildtype zebrafish were cultivated in a 28 °C incubator under a photoperiod of 10D/14L. Zebrafish eggs were maintained in salt water at 28 °C. After 48 hpf, dechorionated eggs were prepared in a Petri dish. To examine the toxicity of the DQA and DTOS emulsions, larvae were incubated with the samples, after which the number of surviving larvae were counted, and the morphology of the larvae was observed. We generated the zebrafish cancer models using the HeLa cell line for biodistribution and anticancer experiments. HeLa cells were stained using a cell fluorescence tracker (CellTracker™ Green CMFDA Dye, Invitrogen, Carlsbad, CA, USA) and injected into the yolk of larvae (48 hpf) using a microinjector (≈100 cells/larva for biodistribution and ≈150 cells/larva for anticancer). To determine the biodistribution, the xenografted larvae were incubated with 20 µM of Nile red-loaded DTOS 5-5 emulsion for 2 days. For the anticancer effect, the xenografted larvae were incubated with 30 µM of Nile red-loaded DTOS 5-5 emulsion for 3 days. After the incubation, the larvae were anesthetized using 0.04% tricaine and observed under a confocal microscope (Zeiss, Oberkochen, Germany).

### 2.15. Statistical Analysis

The results are expressed as mean ± SD. Statistical analysis (*p*-value) was performed using GraphPad Prism 5 (*t*-test and ANOVA). Differences between groups were considered statistically significant at *p* < 0.001 (***).

## 3. Results

### 3.1. Fabrication of DTOS Emulsion

**Figure 3:**
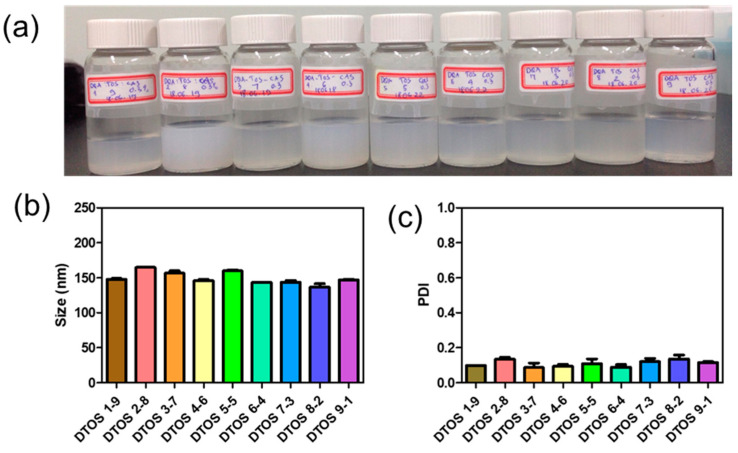
(**a**) Digital photograph of the dequalinium (DQA) and α-tocopherol succinate (α-TOS) (DTOS) emulsions after preparation. (**b**) Dynamic light scattering (DLS)-measured diameter of the DTOS emulsion after preparation. (**c**) Polydispersity index (PDI) of the DTOS emulsion after preparation. The data are presented as the mean value ± standard deviation of triplicate experiments.

Dequalinium and α-TOS formed an emulsion at different molar ratios (Table 1 and Figure 2). Based on the amount of the DQA and α-TOS, we prepared DTOS 1-9, DTOS 2-8, DTOS 3-7, DTOS 4-6, DTOS 5-5, DTOS 6-4, DTOS 7-3, DTOS 8-2, and DTOS 9-1 emulsions. The DTOS emulsions were obtained using a homogenizer, which is effective for generating emulsions with suspended droplets < 200 nm in diameter. All emulsions were formed at nano-size (around 150–170 nm) (Figure 3). The size distribution range was small, as demonstrated by the polydispersity index (PDI) value, where a PDI value < 0.2 represented a monodispersed droplet size distribution. The results implied that we successfully created a homogeneous nanoemulsion containing DQA, α-TOS, and castor oil using a homogenizer. The encapsulation efficiency of DQA and α-TOS was evaluated using a standard curve (Figure S1, Supplementary Materials). DTOS 5-5 was the best ratio for the emulsion formulation with a high DQA and α-TOS encapsulation efficiency (Table 1). The higher the DQA concentration, the higher the encapsulation efficiency of α-TOS. This may be due to the surfactant property of DQA.

### 3.2. Characterization of Emulsion

**Figure 4:**
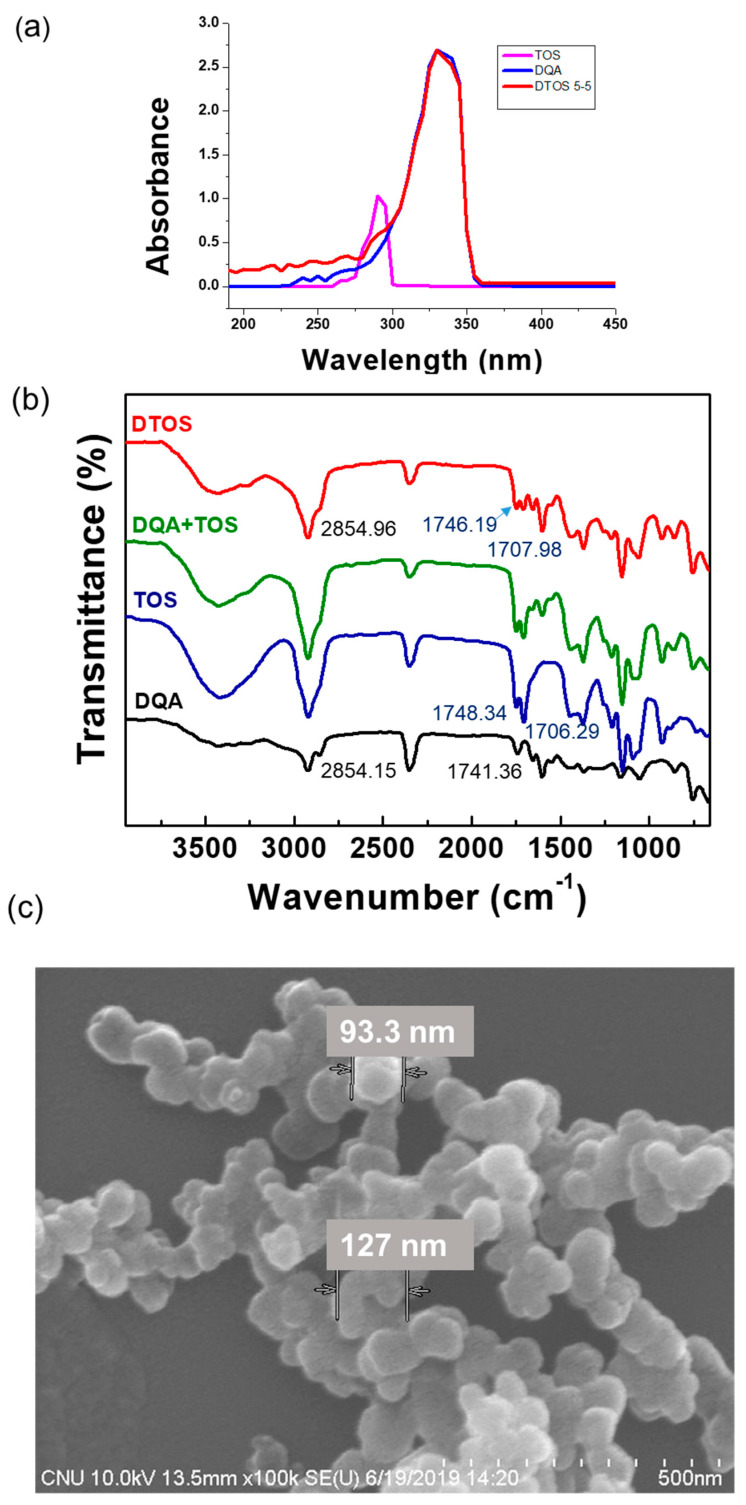
(**a**) UV-Vis absorption spectra of α-TOS, DQA, and DTOS emulsions. (**b**) FTIR spectra of α-TOS, DQA, the mixture of DQA and α-TOS, and DTOS emulsions. (**c**) Field emission scanning electron microscopy of DTOS 5-5.

The confirmation of the emulsion component was evaluated using UV-Vis absorbance. DQA had an absorbance at 330 nm and α-TOS had an absorbance at 290 nm (Figure 4a). The DTOS 5-5 emulsion presented both peaks of α-TOS and DQA. The FTIR results showed the presence of DQA and α-TOS in the emulsion (Figure 4b), providing confirmation of the successful production of the DTOS emulsion. The shape, size, and surface charge of the nanoparticle are important factors for nanomedicine. The spherical nanoemulsion was detected using field emission scanning electron microscopy (Figure 4c). The DQA and DTOS 5-5 emulsion sizes were 208.4 ± 3.36 and 155.8 ± 0.9, which were smaller than the control IPM emulsion (435.67 ± 30.17 nm). The surface charges of the emulsions were determined: the control emulsion had a negative charge (−4.2 ± 0.4 mV), and the DQA and DTOS 5-5 emulsions had a positive charge (42.77 ± 3.34 and 34.4 ± 4.2) (Table 2). The positive charge of the emulsion may enhance its binding to cancer cells, facilitating intracellular targeted delivery [36]. These data are promising for cancer treatment. Nanoemulsions can easily target the tumor side due to the enhanced permeability and retention effect. The positive charge around the DTOS 5-5 emulsion facilitates interaction with the cell membrane.

### 3.3. Stability of Emulsion

**Figure 5:**
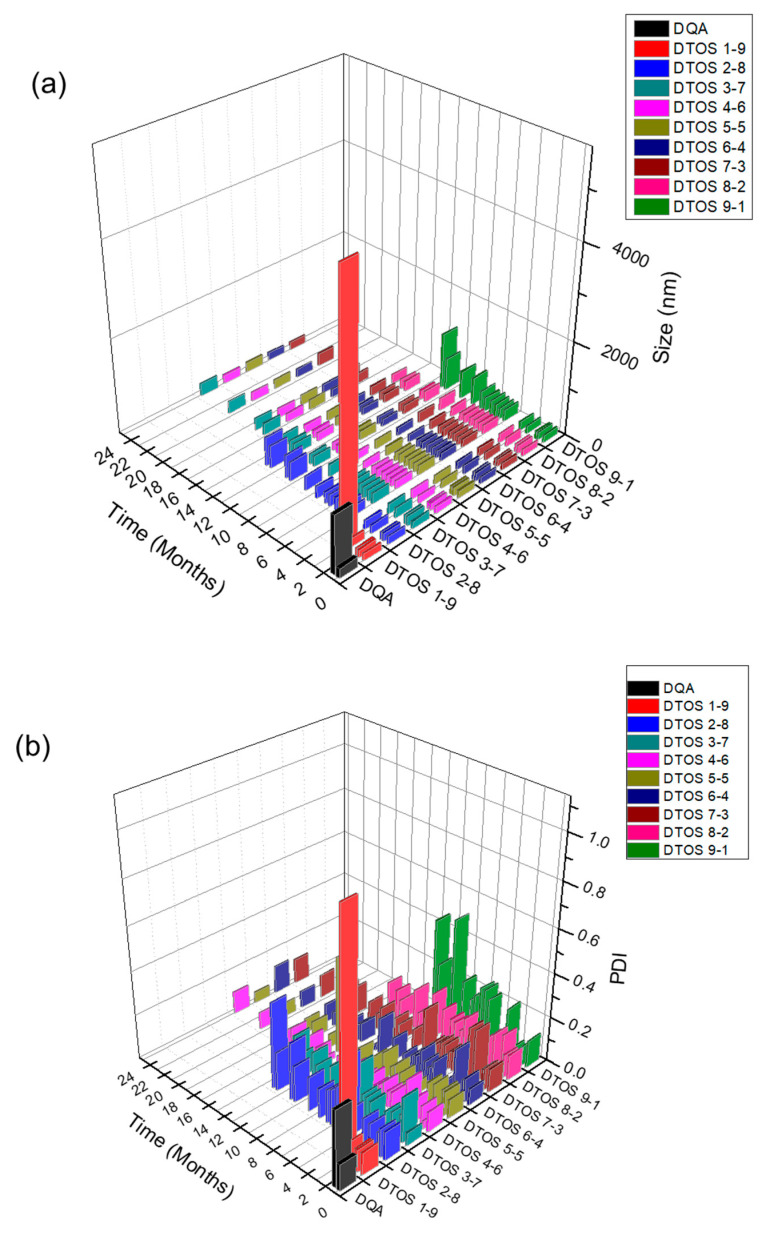

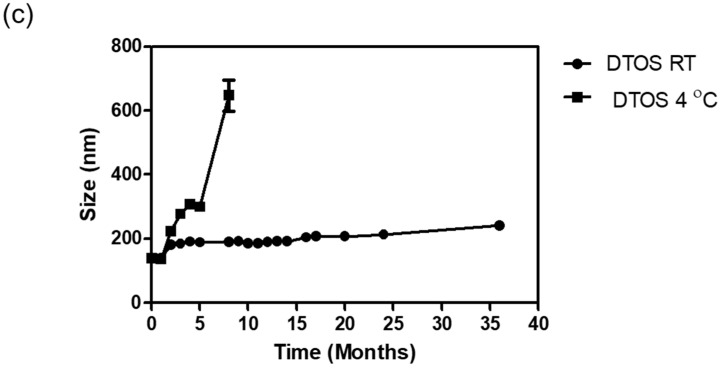
The stability of the DTOS emulsion. (**a**) Mean DLS diameter of the DTOS emulsion at a defined time. (**b**) Mean polydispersity index of the DTOS emulsion at a defined time. (**c**) DTOS emulsion stability under different temperatures at a specified time. The data are presented as the mean value ± standard deviation of triplicate experiments.

The emulsions were stored at room temperature. The size was confirmed at a certain time using an ELS-Z particle size analyzer (Otsuka Electronics, Otsuka, Japan). Figure 5 shows the stability of the emulsions depending on the amount of the DQA and α-TOS. The emulsion containing only DQA and castor oil (DQA emulsion) was not stable, and its size changed after 2 weeks, and the PDI value also increased. This indicates that the DQA emulsion cannot avoid the aggregation of oil droplets after forming nanoparticles. α-TOS plays an important role of a co-surfactant in stabilizing the emulsion, which is a good indicator for the application of our emulsion. The sizes of DTOS 3-7, DTOS 4-6, DTOS 5-5, DTOS 6-4, and DTOS 7-3 emulsions were stable for over two years at room temperature (Figure 5a). However, the PDI value of DTOS 5-5 was the most stable (Figure 5b). Therefore, the DTOS 5-5 emulsion was selected for further experiments. The HeLa cells were tested for the anticancer effect of DTOS 5-5 when fresh and after storage at room temperature for 1 and 3 months. Figure S2 revealed that the bioactivity was consistently similar, implying the stability of the emulsion. However, the DTOS 5-5 emulsion was not stable at 4 °C (Figure 5c). The DQAsome has been demonstrated to be unstable under low temperatures. The mechanism of emulsion stability has been unclear until now. The combination of DQA and α-TOS increased the stability of the emulsion, leading to improved potential in nanomedicine applications.

### 3.4. Nile Red-Loaded Emulsion, Cell Uptake, and Mitochondria-Targeting

**Figure 6:**
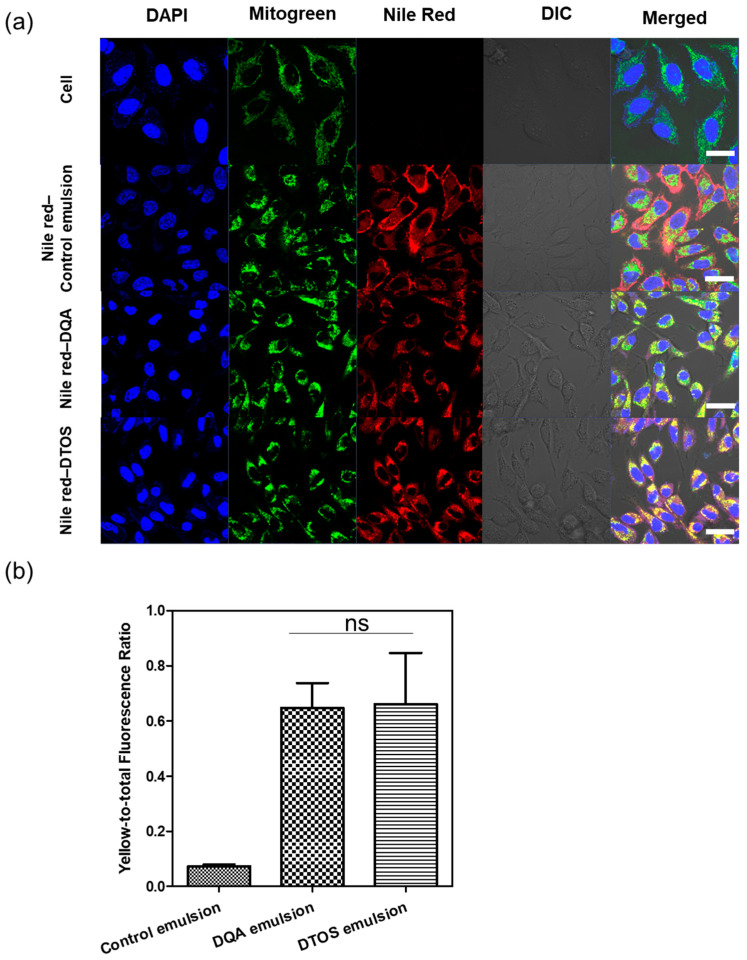
(**a**) Confocal laser scanning microscopy images of HeLa cells after 24 h of incubation with the Nile red emulsion. Labeled nanoparticles are shown in red (Nile red). The mitochondria are stained with MitoGreen. The cell nucleus is stained blue by 4′,6-diamidino-2-phenylindole (DAPI). DIC, differential interference contrast; DQA, dequalinium emulsion; DTOS, DQA/α-TOS emulsion. The scale bar represents 30 µm. (**b**) The DTOS emulsion showed significant colocalization with mitochondria comparable to the DQA emulsion. Data points are represented as mean ± SD (n = 3 images for each group). Statistical analysis was conducted using *t*-test. ns: not significant. The scale bar represents 30 µm.

Nile red was used as a fluorescence model and was loaded into the emulsions and characterized. The Nile red emulsion was incubated with HeLa cells for 24 h. Mitochondria were stained with mitogen. The nucleus was stained with Hoechst dye and imaged using confocal laser scanning microscopy (Figure 6). The red fluorescence showed the location of the emulsion and the nanoemulsion produced a high-intensity red color, indicating that the emulsion can be taken up inside the cell membrane. The positive surface charge of nanoemulsion supports that it can effectively interact with the cell membrane, which is the first step of the endocytosis pathway. In addition, the Nile red emulsion can be localized at the mitochondria, where we can find the yellow color in the cells. The DQA and DTOS emulsions were both found to be able to efficiently target the mitochondria.

### 3.5. Mitochondrial Membrane Potential

**Figure 7:**
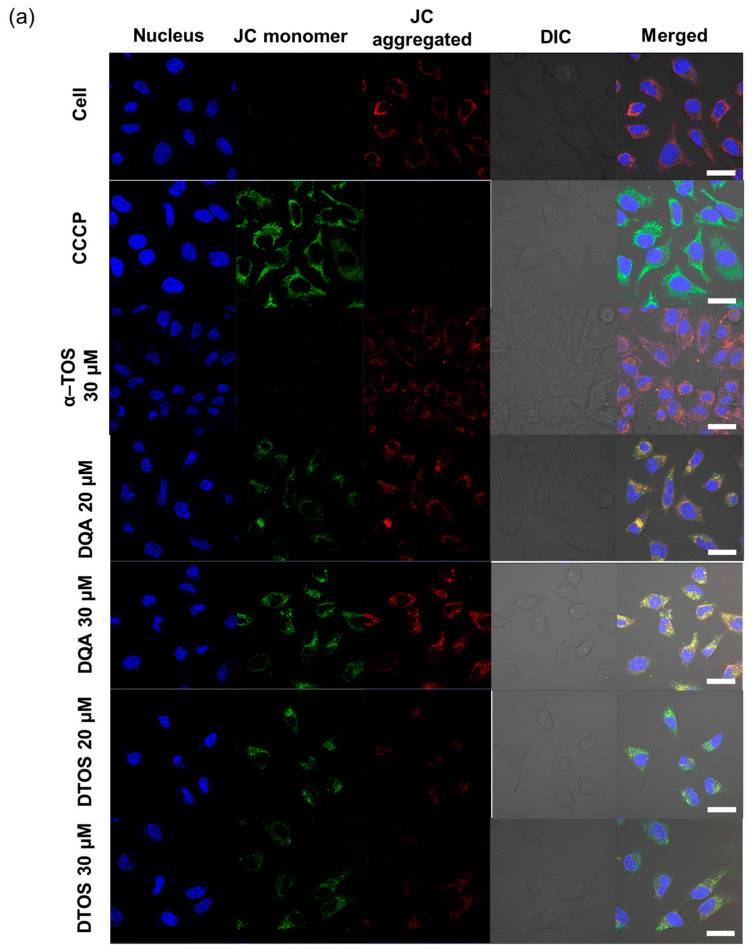

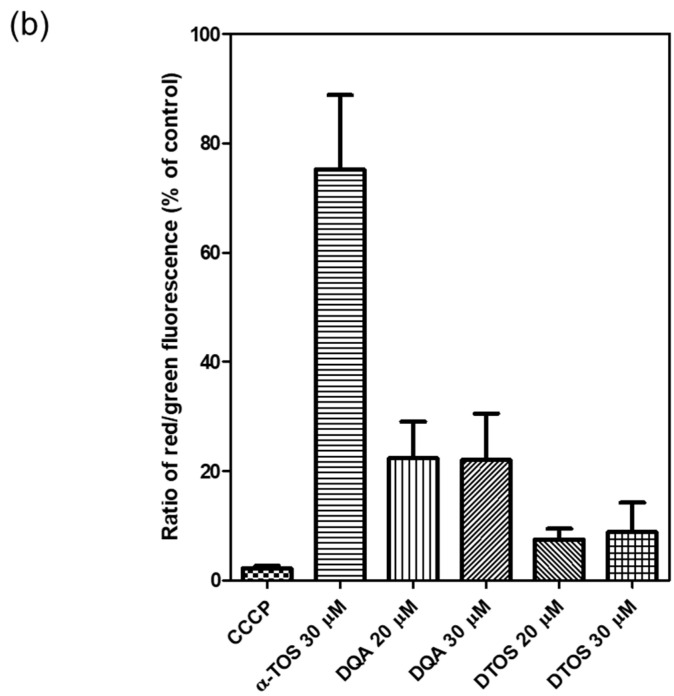
Assay of HeLa cell mitochondrial membrane potential with the JC-1 probe staining method. (**a**) Depolarization of mitochondrial membrane potential was observed after the cells were exposed to the emulsion, for 24 h, with CCCP as the positive control. (**b**) Quantitative analysis of the ratio of red/green fluorescence intensity. Results of the red and green fluorescence are overlapped and expressed as the mean ± standard deviation of triplicates. DIC, differential interference contrast; DQA, dequalinium emulsion; α-TOS, α-tocopherol succinate; DTOS, DQA/α-TOS emulsion. The scale bar represents 30 µm.

HeLa cells were treated either with the DQA emulsion or the DTOS 5-5 emulsion. Mitochondrial oxidative phosphorylation uncoupler (CCCP), which has low potential, was used as a positive control. Then, the fluorescent probe JC-1 was added. The data showed a decreased red (~590 nm) to green (~529 nm) fluorescence intensity ratio in response to both DQA and DTOS emulsion exposure in relation to mitochondria depolarization, which is clearly observable in the fluorescence images. The results also showed that the impact of the DQA emulsion was less than that of the DTOS emulsion (Figure 7).

### 3.6. Cell Viability

**Figure 8:**
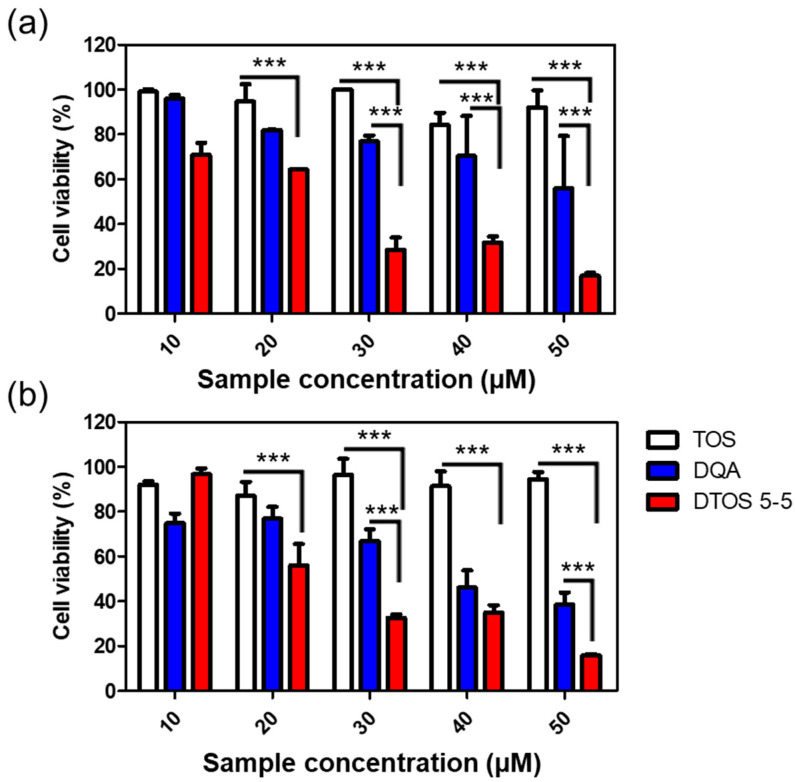
Cell viability of α-TOS, DQA, and DTOS 5-5 emulsions at different concentrations measured in HeLa cells at (**a**) 24 and (**b**) 48 h. Data points are represented as mean ± SD of four individual copies. Statistical analysis of data was conducted by one-way ANOVA with Tukey’s post-hoc test. Differences between groups were considered statistically significant at *p* < 0.001 (***).

The cytotoxicity effects of DTOS 5-5 were evaluated in HeLa cells using a WST-1 assay. Figure 8 shows that the DQA and DTOS 5-5 emulsions had an anticancer effect on the HeLa cells. Moreover, the DTOS emulsion caused toxicity in the B16F10 and MCF-7 cells (Figure S3). Previous studies have shown that DQA has the ability to inhibit cancer cell growth [25]. Dequalinium has a positive charge and hydrophobic head. It may cause a high interaction with the cell membrane and destroy the stability of the cell membrane. In other words, the accumulation of a high concentration of DQA inside the mitochondria can produce impaired mitochondria. In addition, the presence of α-TOS can improve the accumulation of DQA, increasing the impairment of the mitochondria and even causing cell death.

### 3.7. 3D Spheroid

**Figure 9:**
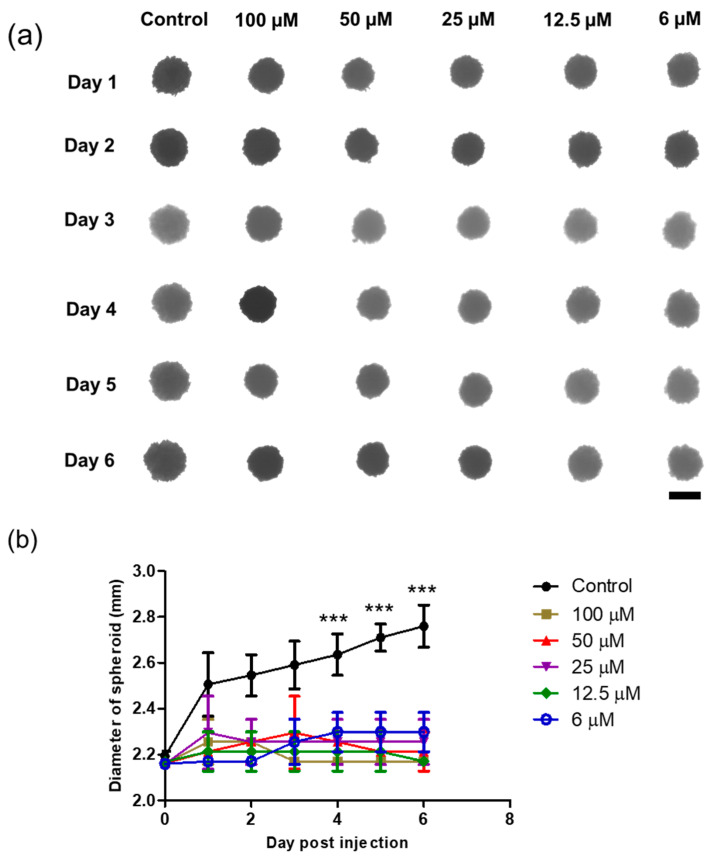
Three-dimensional spheroid study of the DTOS 5-5 emulsion. (**a**) Morphology and (**b**) diameter of spheroids. The scale bar represents 2.30 mm. A significant difference was observed between the emulsion treatment group (25 µM) and the control group. Data points are represented as mean ± SD of three individual copies. Statistical analysis of data was conducted using *t*-test (***, *p* < 0.001).

A 3D spheroid model was formed by seeding HeLa cells in agarose-coated 96-well plates, and single spheroids were successfully grown for 3 days before sample treatment. The spheroids acquired a spheroid shape and became darker over time owing to the increase in the cell population. The morphology and size of the spheroids were monitored, followed by sample treatment. Interestingly, as shown in Figure 9, the DTOS 5-5-exposed spheroid showed peripheral cell damage. Compared to a 2D culture, where DTOS 5-5 effects are noticeable within 24 h of treatment, 3D models provide a closer insight into tumors where drug penetration and availability are limited due to their dense core and rigid extracellular matrix. These results suggest that 3D organization and tissue formation make tumors more resistant and predominantly complex compared to 2D culture models when therapeutic drugs are tested.

### 3.8. Zebrafish Animal Experiments

**Figure 10:**
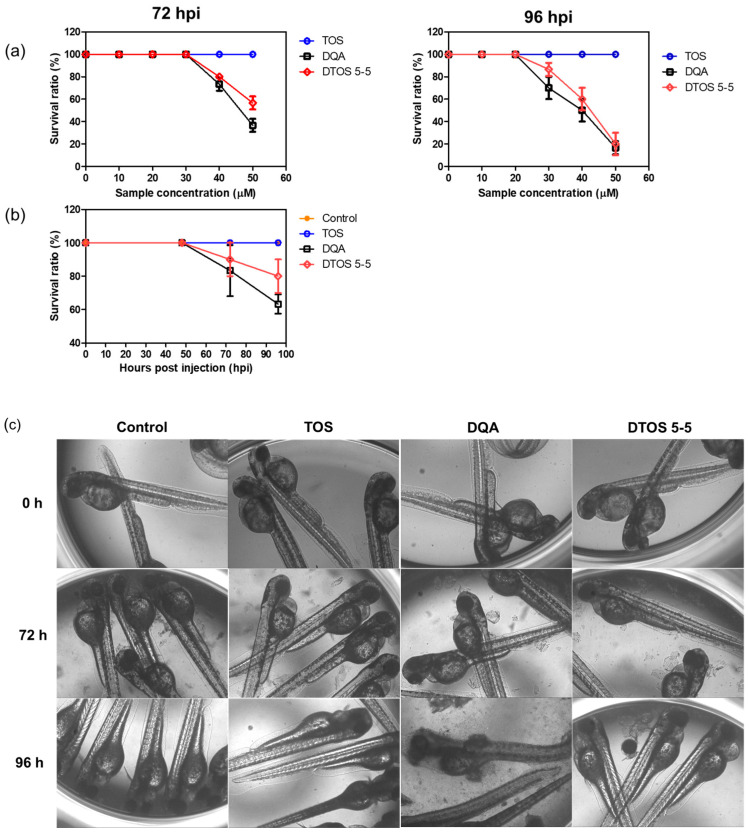
Evaluation of the in vivo toxicity of dequalinium (DQA) and α-tocopherol succinate (α-TOS) (DTOS) emulsions in zebrafish larvae. (**a**) The survival ratio of zebrafish larvae after treatment with various concentrations of samples at 72 and 96 hpi. Each value represents the average and standard deviation (n = 3). (**b**) The survival ratio of zebrafish larvae after treatment with 30 µM at a certain time. Each value represents the average and standard deviation (n = 3). (**c**) The morphology of the embryos after treatment with 30 µM of samples at a certain time. hpf, hours post-fertilization.

**Figure 11:**
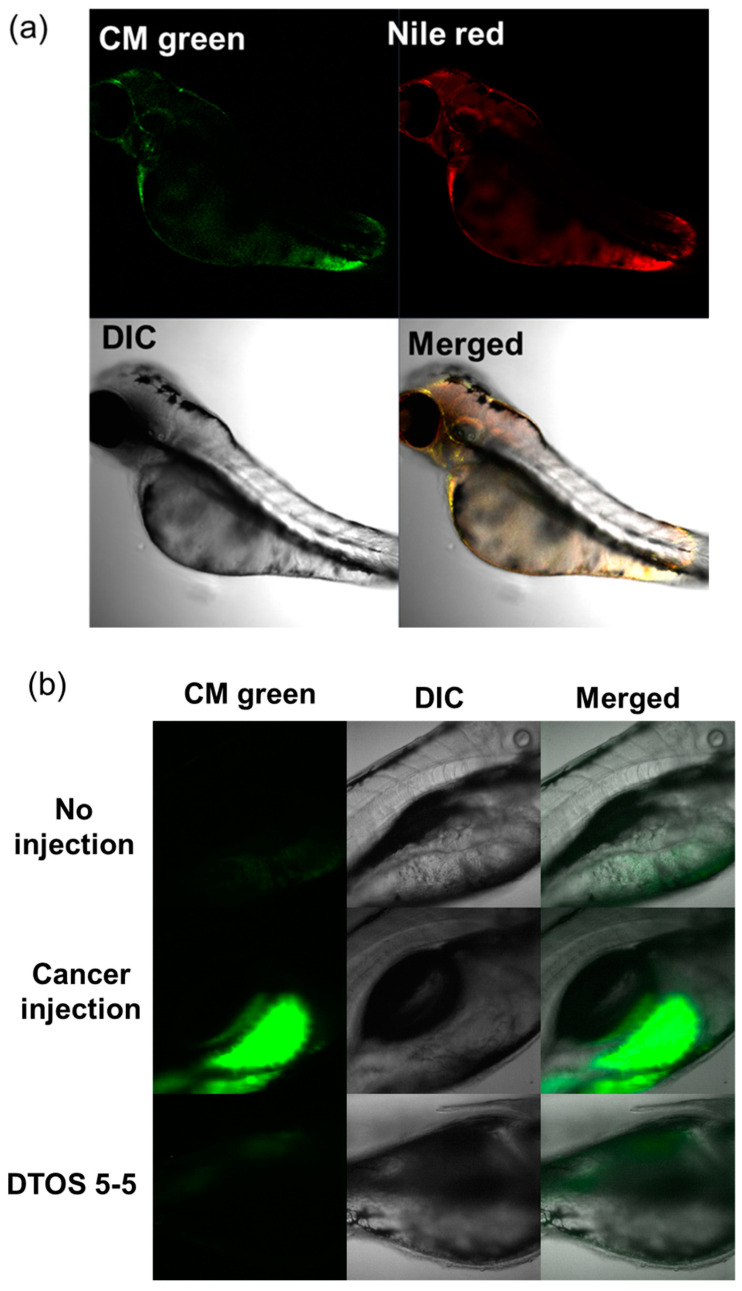

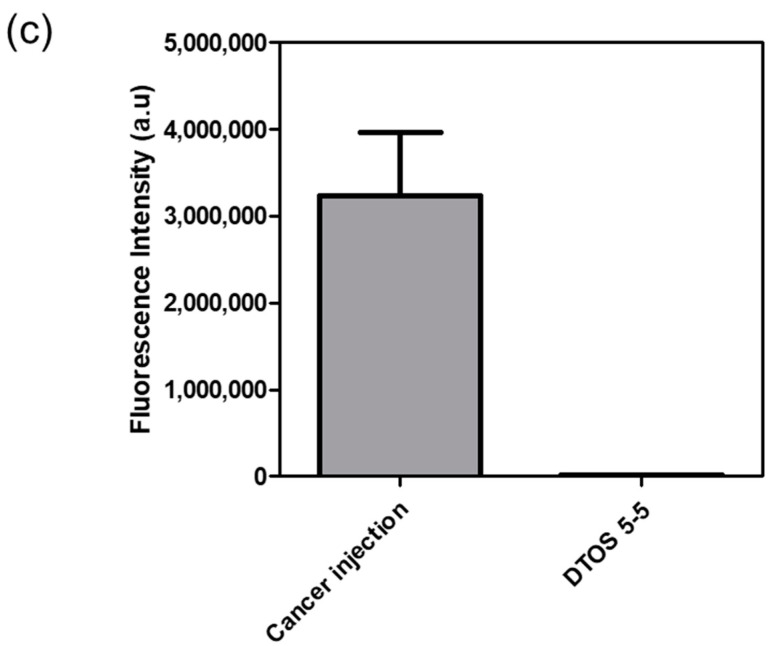
HeLa cells xenografted onto zebrafish larvae. (**a**) Colocalization of the emulsion and cancer cells. (**b**) Anticancer effect of the emulsion on the HeLa cells xenografted onto zebrafish larvae. (**c**) Fluorescence intensity of cell-tracker-labeled HeLa cells in zebrafish larvae. Each value presents the average and standard deviation (n = 3). DIC, differential interference contrast; hpf, hours post-fertilization; hpi, hours post-injection.

Human cancer cell xenografts in zebrafish larvae are a helpful preclinical tool in oncology research. Before evaluating the anticancer effect of the DTOS 5-5 emulsion, we examined its toxicity on the larvae. The larvae (48 h post-fertilization (hpf)) were incubated with various concentrations of the samples (10 µM to 50 µM) and observed at 72 and 96 h post-injection to record the number of alive larvae. α-TOS and DQA were used as the controls. Figure 10a showed that the α-TOS was safe for larvae even though they were treated at a high concentration. However, the DTOS 5-5 emulsion caused toxicity for larvae owing to the addition of DQA. The positive charge may interact with the fish, causing death. DTOS 5-5 doses of 10–30 µM were found to be safe for larvae. Moreover, DTOS 5-5 showed an anticancer effect at 30 µM in vitro. Therefore, we selected this concentration for further study. DTOS 5-5 at 30 µM was used to evaluate the toxicity of larvae for a certain time. The survival ratio of larvae was 80% at 96 dpi, while the DQA caused more toxicity. The morphology of the larvae was evaluated. We performed confocal laser scanning microscopy analysis to determine the biodistribution of the cancer cells and emulsion in the zebrafish larvae. We observed the HeLa cells as green and the emulsion as red. Figure 11a indicates that the cancer cell grew well on the larvae. The emulsion became distributed throughout the fish’s body. The yellow color in the merged picture indicates the colocalization of cells and emulsions. The anticancer effect was then evaluated with DTOS 5-5, 30 µM. After the xenografted model was successfully prepared, the xenografted larvae were incubated with the DTOS 5-5 emulsion for 3 days. The local cancer cells were captured. Figure 11b shows that the DTOS 5-5 emulsion inhibited the growth of the HeLa cells on the larvae. The fluorescence intensity of the cell tracker-labeled HeLa cells in the zebrafish larvae was analyzed using Image J. Less fluorescence was found in the case of the DTOS 5-5 emulsion compared to that in the controls (Figure 11c), demonstrating the strong anticancer effect of the DTOS 5-5 emulsion.

## 4. Discussion

Castor oil, which has been used as folk medicine since Ancient Egyptian times [37], was chosen as the oil phase. The castor oil is hydrolyzed in the small intestine by pancreatic enzymes, which results in the release of glycerol and ricinoleic acid, although 3,6-epoxyoctanedioic acid, 3,6-epoxydecanedioic acid, and 3,6-epoxydodecanedioic acid also appear as metabolites [38]. Castor oil and ricinoleic acid easily penetrate deep into the skin and enhance the transdermal penetration of other chemicals [39]. Among the different mitochondria-targeting moieties, DQA is suitable for emulsion emulsifiers due to its amphiphilic property. Moreover, α-TOS as a co-component can improve the effects of anticancer treatment. It has numerous important roles in the body and cells because of its antioxidant activity. Oxidants have been linked to numerous possible conditions and diseases, including cancer.

Herein, we explored whether the combination of castor oil, DQA, and α-TOS is suitable for anticancer effects. The stability of the emulsion is related to the concentration of DQA and α-TOS. We found that the range of each component from 3 mM to 7 mM is perfect for the stability of the DTOS emulsion at RT (Table 1 and Figure 5). α-TOS contributed to this characterization of the emulsion. The DQA emulsion was found unstable (data not shown). The stability mechanism is still ambiguous. We hypothesized that it is related to the surfactant ability of DQA. The size of the emulsion is small, which facilitates passing the enhanced permeability and retention (EPR) effect to reach the tumor site (Figure 3). The positive charge of the emulsion is good to interact with cell membranes (Table 2); however, it may be a disadvantage of our emulsion due to the interaction with plasma protein. The surface modification of the DTOS emulsion is needed in further studies. The cell uptake data showed that the DTOS emulsion can be located in the mitochondria (Figure 6). Moreover, mitochondria membrane potential has been considered the master signal between cell death and survival. Lipophilic cations can accumulate inside the inner membrane of the mitochondria and reduce the membrane potential. Previous reports have shown that anti-apoptotic signals from the cancer cell arise from the impairment of the mitochondrial membrane potential [40]. Therefore, to understand the mechanism of cell death related to mitochondria, the mitochondrial membrane potential needs to be confirmed. The JC-1 probe can be used to evaluate the mitochondrial potential. When the membrane potential is low, the JC-1 probe is not taken up by the mitochondria and is present in the cytoplasm as a fluorescent green monomer rather than being taken up and forming a fluorescent red aggregate when the mitochondrial membrane potential is intact. Our data showed that the mitochondrial membrane potential was low in the case of the DQA and DTOS emulsions (Figure 7). This may be a cause of cell death. In addition, the cell viability results demonstrated cell death after being treated with the DTOS emulsion (Figure 8).

The 3D tumor spheroids are universally used for drug screening and have recently obtained noticeable attention in cancer therapy for the study of cancer cell-to-cell communication, invasion, and drug resistance mechanisms [41]. Three-dimensional spheroid tumor models reportedly have better potential to mimic in vivo tumor environments than 2D culture models; therefore, we modeled the anticancer effects of DTOS 5-5 in a 3D tumor spheroid (Figure 9 and Figure S4). In 2D models, cancer cells are equally exposed to cytotoxic drugs, which do not sufficiently mimic the complexity of in vivo tumors. In contrast, 3D tumor models possess several features, such as drug penetration, hypoxia, and an extracellular matrix, that could shorten the gap between 2D testing and animal models. For the in vivo application, the zebrafish larvae were used as a model to evaluate the ability of the emulsion on anticancer effects. The cancer cells were xenografted on the larvae and then treated with the emulsion. The DTOS emulsion can enter and distribute inside the zebrafish larvae and inhibit cancer cell growth (Figure 11).

## 5. Conclusions

Here, we reported the fabrication of a mitochondria-targeting emulsion containing DQA and α-TOS. The resulting nanoemulsion, termed DTOS, was a homogenous emulsion shown to be 150–170 nm in diameter and stable for a long time at room temperature. After testing different ratios, the DTOS 5-5 emulsion was chosen as the agent for further study. The DTOS 5-5 emulsion can target mitochondria and reduce the mitochondrial membrane potential. It can inhibit the cell proliferation of cancer in both 2D and 3D models, as well as in cell xenografts on zebrafish larvae. Our results highlight that a straightforward combination of DQA and α-TOS is a beneficial combination for emulsion formulation that should be further researched for its positive potential in nanomedicine applications.

## Figures and Tables

**Table 1:** Encapsulation efficiency of DQA and α-TOS emulsions.

	DQA (mM):α-TOS (mM):Castor Oil (%)	DQA Encapsulation Efficiency (%)	α-TOS Encapsulation Efficiency (%)
DTOS 1-9	1:9:3	98.7	11.7
DTOS 2-8	2:8:3	89.1	36.8
DTOS 3-7	3:7:3	54.9	28.2
DTOS 4-6	4:6:3	58.8	43.3
DTOS 5-5	5:5:3	99.6	99.6
DTOS 6-4	6:4:3	75.2	91.9
DTOS 7-3	7:3:3	96.4	96.9
DTOS 8-2	8:2:3	83.8	99.9
DTOS 9-1	9:1:3	71.7	99.1

**Table 2:** Size and zeta potential of dequalinium (DQA) emulsion and α-TOS/DQA 5-5 emulsion (DTOS 5-5).

	Zeta Potential (mV)	Size (nm)	PDI
Control emulsion	−4.23 ± 0.37	435.67 ± 30.17	0.39 ± 0.01
DQA	42.77 ± 3.34	208.40 ± 3.36	0.17 ± 0.01
DTOS 5-5	34.40 ± 4.22	155.83 ± 0.85	0.15 ± 0.01

## Data Availability

Data are contained within the article and the Supplementary Materials.

## Supplementary Materials

The following supporting information can be downloaded at: https://www.mdpi.com/article/10.3390/antiox12020437/s1. DQA and α-Tos encapsulation efficiency. Figure S1: The standard curve of (a) DQA at 330 nm, (b) DQA at 290 nm and (c) α-TOS at 290 nm.; Figure S2: Measurement of HeLa cell viability after 24 hours of exposure to 30 µM DTOS 5-5 emulsion stored at room temperature for the specified period.; Figure S3: Cell viability of DTOS 5-5 emulsion at varying concentrations measured in MDA-MB-231, MCF-7, B16F10 cells at (a) 24 and (b) 48 h; Figure S4: The magnification of the spheroid after treatment with DTOS emulsion on day 6.
