# Supplementary material for: Nanoemulsion Composed of α-Tocopherol Succinate and Dequalinium Shows Mitochondria-Targeting and Anticancer Effects
Source: Antioxidants (Basel). 2023 Feb 10;12(2):437. doi: 10.3390/antiox12020437 (PMC9952168; doi:10.3390/antiox12020437)
Supplement: Supplementary file 1 [file antioxidants-12-00437-s001.zip › antioxidants-2188764-supplementary.pdf]

Nanoemulsion composed of α-Tocopherol succinate and Dequalinium shows mitochondria targeting and anticancer effect

Le Thi Thuy [1], Seulgi Lee [2], Viet Dongquoc [3] and Joon Sig Choi [1,*]

[1]Department of Biochemistry, Chungnam National University, 99 Daehak-ro, Yuseong-gu, Daejeon 34134, Republic of Korea

[2]KM Science Research Division, Korea Institute of Oriental Medicine, Yuseong-daero 1672, Yuseong-gu, Daejeon 34054, Republic of Korea

[3]Department of Materials Science and Engineering, Chungnam National University, 99 Daehak-ro, Yuseong-gu, Daejeon 34134, Republic of Korea

*Correspondence: joonsig@cnu.ac.kr; Tel.: Tel: +82-42-821-7528, Fax: +82-42-822-7548

Supporting information

DQA and α-Tos encapsulation efficiency

The concentrations of DQA and α-Tos in the emulsion were analyzed using an ultraviolet spectrometer (UV-vis). A calibration curve was established for each of the compounds, with DQA being analyzed at wavelengths 330 nm and 290 nm and α-Tos being analyzed at wavelength 290 nm (Figure S1). The high correlation coefficients (R^2 = 0.9954, 0.9983 and 0.9950) obtained from the standard curve fitting confirmed the accuracy of the results. To measure the absorbance of α-Tos in the emulsion, the freeze-dried sample was first dissolved in methanol, vortexed for 1 minute, and then analyzed using UV detection. The absorbance was calculated using the equation provided:

Absorbance of α-Tos $_{290nm}$ = Absorbance of emulsion$_{290nm}$ – Absorbance of DQA$_{290nm}$ (determined from its concentration calculated using its absorbance at 330 nm)

The encapsulation efficiency of DQA and α-Tos was calculated as the following equation, respectively:

$$\text{Encapsulation efficiency (EE\%)} = \frac{Amount\ of\ drugs\ loaded\ in\ emulsion}{Amount\ of\ the\ feeding\ drugs} \times 100\%$$

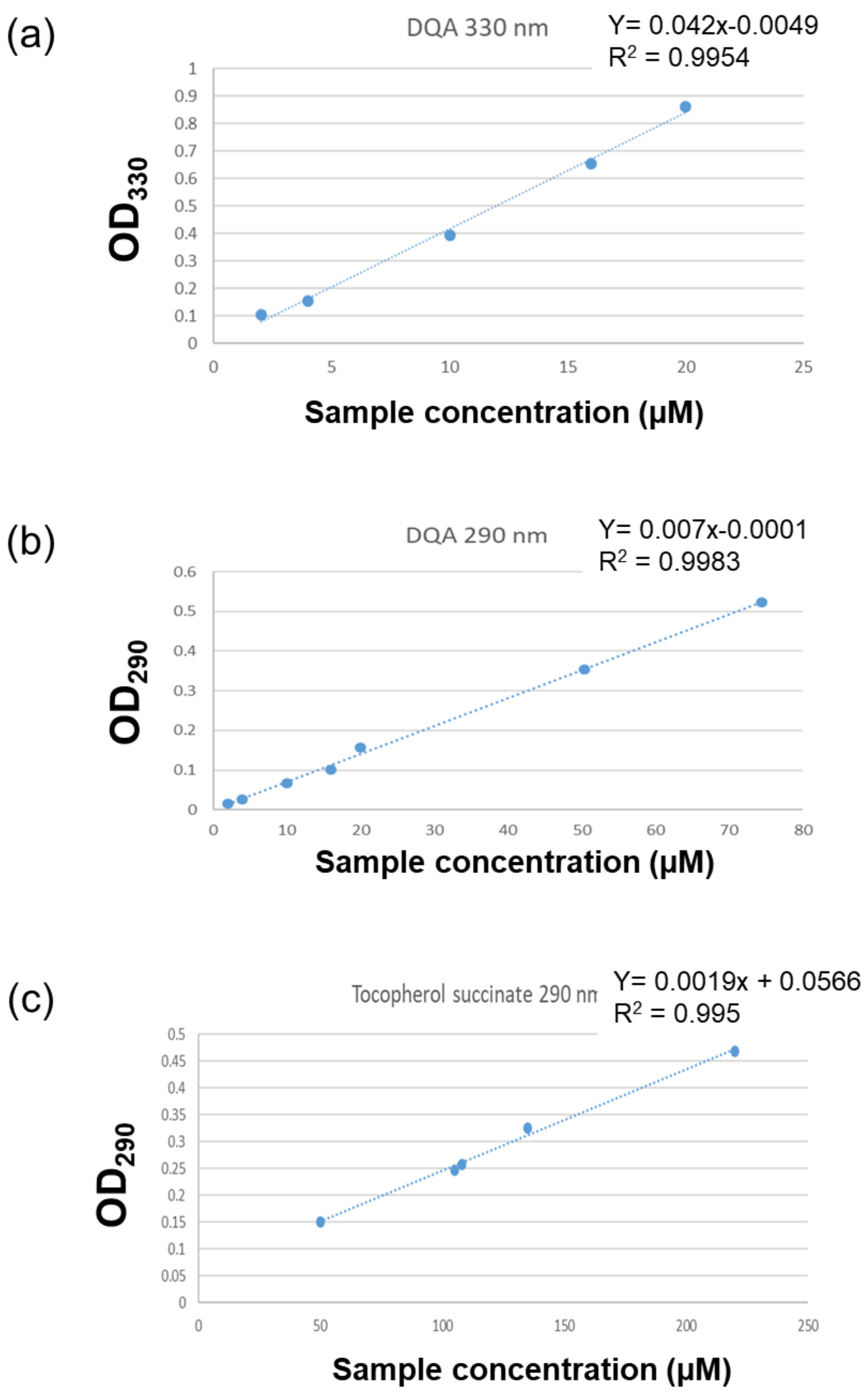

Figure S1. The standard curve of a) DQA at 330 nm, b) DQA at 290 nm and c) α-TOS at 290 nm.

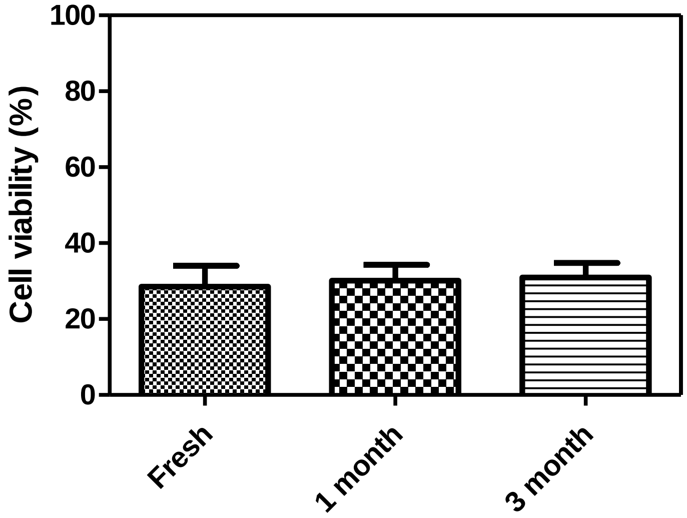

Figure S2. Measurement of HeLa cell viability after 24 hours of exposure to 30 μM DTOS 5-5 emulsion stored at room temperature for the specified period.

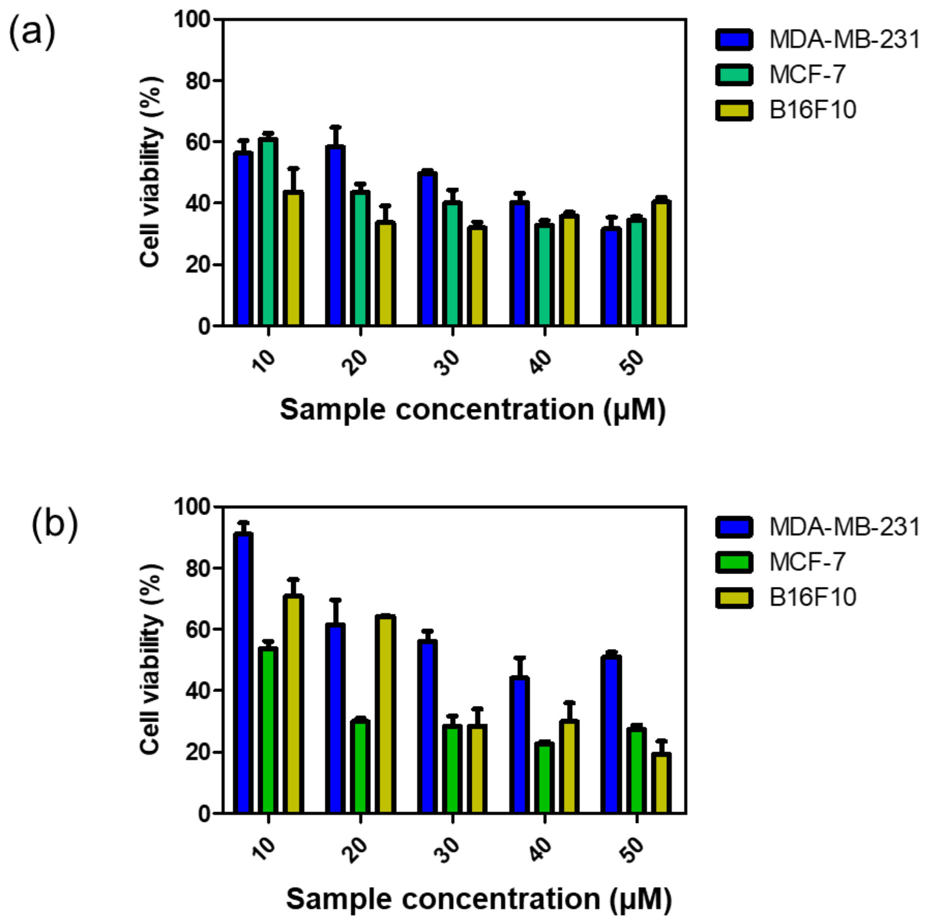

Figure S3. Cell viability of DTOS 5-5 emulsion at varying concentrations measured in MDA-MB-231, MCF-7, B16F10 cells at a) 24 and b) 48 h.

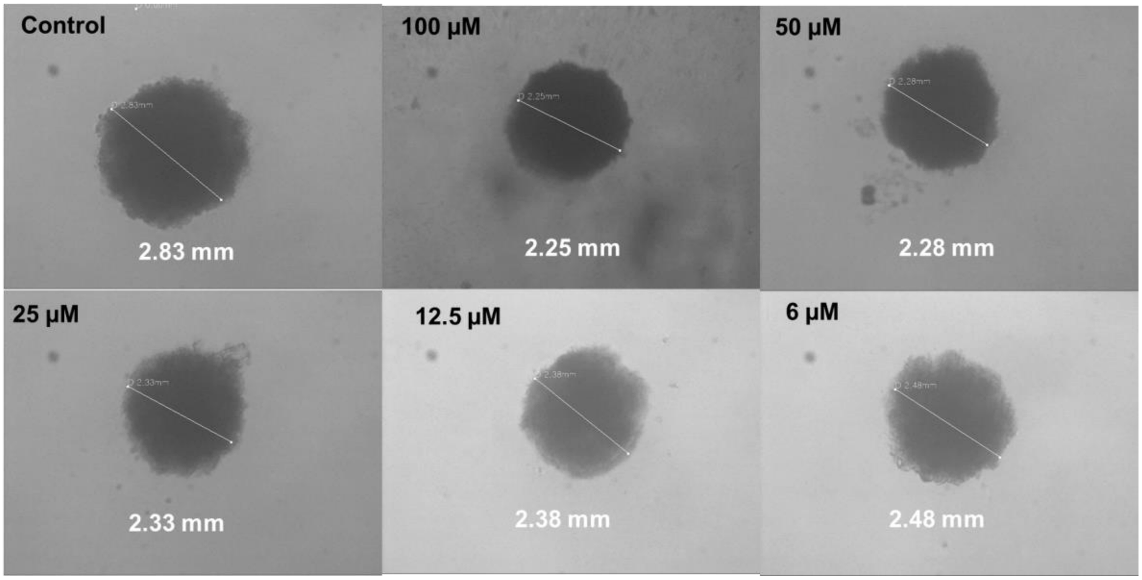

Figure S4. The magnification of the spheroid after treatment with DTOS emulsion on day 6.
